# Use of a novel re-openable endoclip for the closure of a large mucosal defect after endoscopic submucosal dissection

**DOI:** 10.1055/a-2109-0883

**Published:** 2023-07-13

**Authors:** Daiki Kitagawa, Satoki Shichijo, James W. Li, Yuki Okubo, Yoji Takeuchi, Noriya Uedo

**Affiliations:** 1 Department of Gastrointestinal Oncology, Osaka International Cancer Institute, Osaka, Japan; 2 Department of Gastroenterology, Osaka Metropolitan University Graduate School of Medicine, Osaka, Japan; 3 Department of Gastroenterology and Hepatology, Changi General Hospital, Singapore; 4 Department of Gastroenterology and Hepatology, Gunma University Graduate School of Medicine, Maebashi, Japan

A 71-year-old woman with a 100-mm granular type laterally spreading tumor of the sigmoid colon was referred to our hospital ( Fig. 1 ). Endoscopic submucosal dissection (ESD) with en bloc resection of the tumor was performed in 89 min. The specimen measured 105 × 65 mm, and the post-ESD mucosal defect occupied more than three-quarter of the luminal circumference ( Video 1 , Fig. 2 ). We closed the large mucosal defect along its long axis using a novel re-openable endoclip (Mantis Clip; Boston Scientific, Natick, Massachusetts, USA) to minimize adverse events and prevent stricture formation ( Fig. 3 ).

**Fig. 1:**
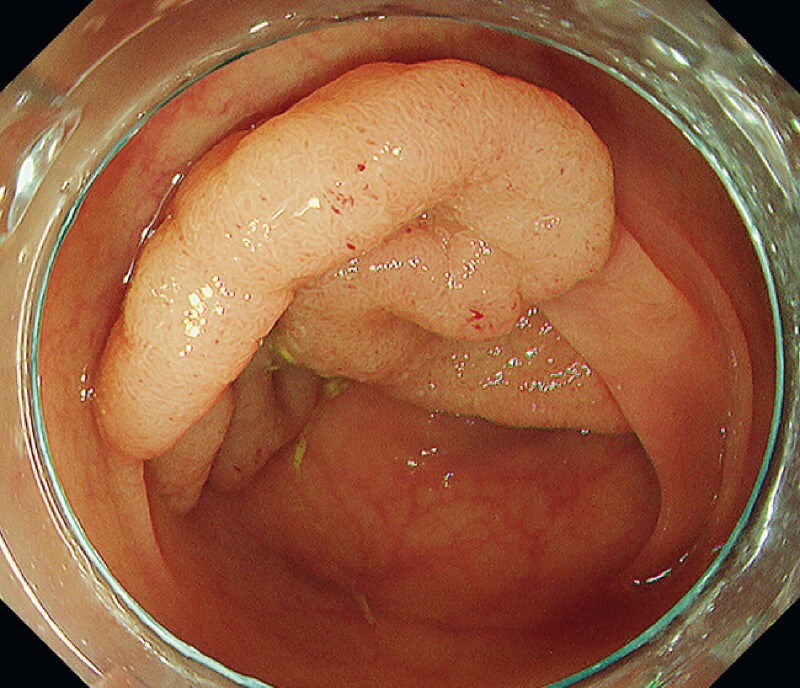
Granular type laterally spreading tumor (100 mm) of the sigmoid colon.

**Video 1** Use of a novel re-openable endoclip for closure of a large mucosal defect that formed during endoscopic submucosal dissection of a laterally spreading tumor.


**Fig. 2:**
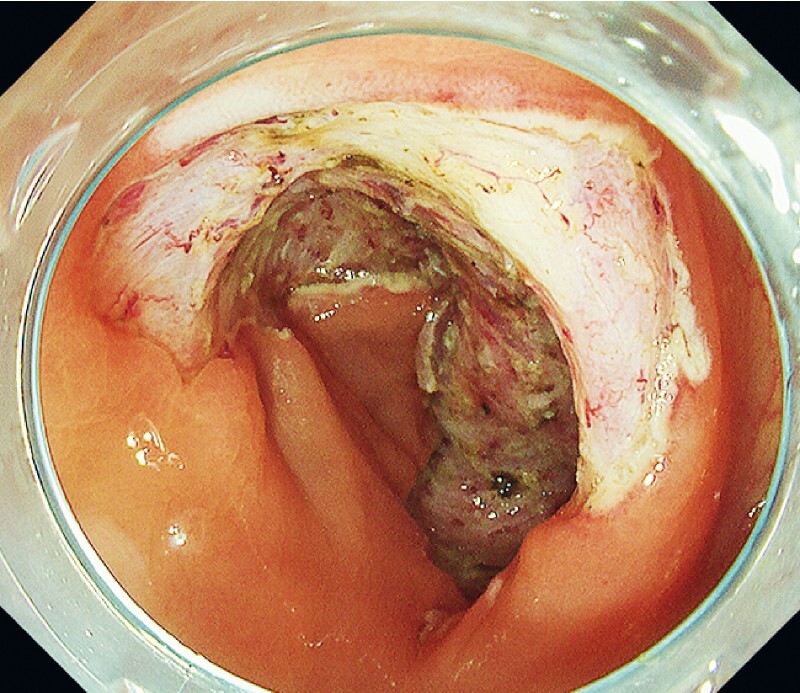
Following endoscopic submucosal dissection, a mucosal defect occupying over three-quarters of the luminal circumference is seen.

**Fig. 3:**
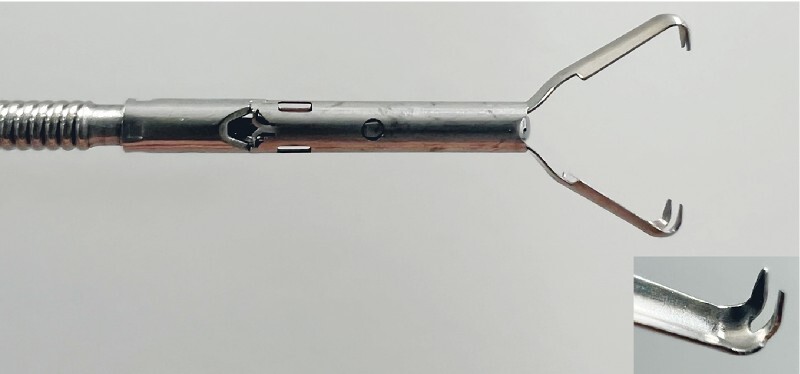
Re-openable endoclip (Mantis Clip; Boston Scientific, Natick, Massachusetts, USA) with TruGrip anchor prongs that prevent slippage of mucosal edge.

First, the distal edge of the mucosal defect was grasped with the re-openable endoclip. Then, the endoscope was inserted into the proximal edge of the mucosal defect, and the endoclip was re-opened. The anchor prongs on the open jaw of the endoclip prevented slippage of the distal edge of the mucosa, which facilitated the grasping of the edges along the long axis of the mucosal defect. Using this method, a single clip was used to appose the widest part of the mucosal defect ( Fig. 4 ). Additional conventional clips were placed until the defect was closed ( Fig. 5 ). The patient was discharged and did not experience any adverse events.

**Fig. 4:**
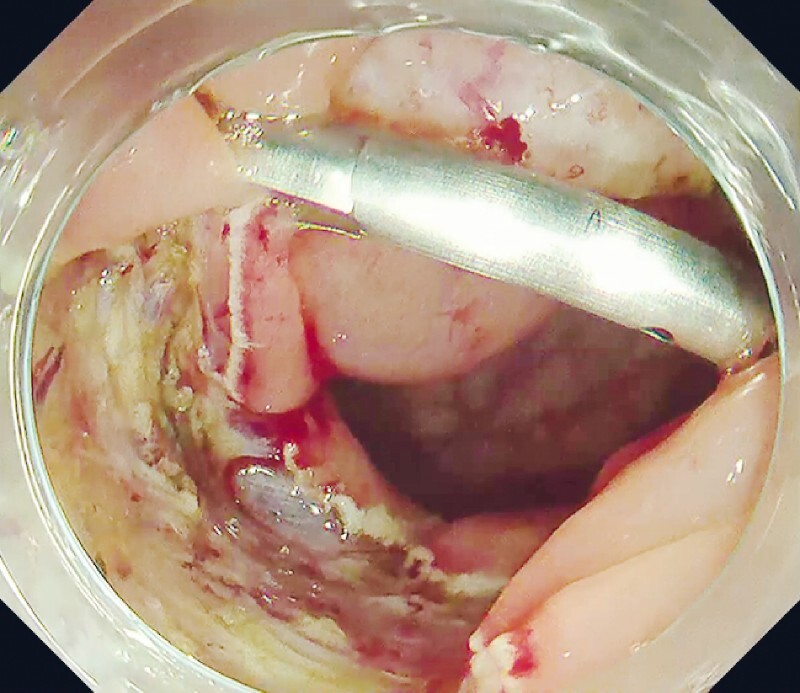
Central part of the large mucosal defect is closed using a single clip.

**Fig. 5:**
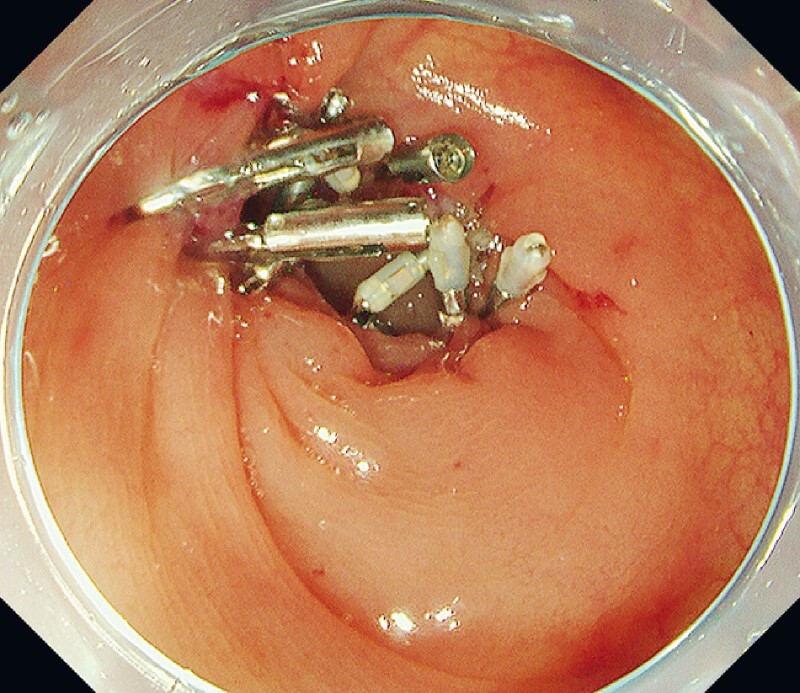
Placement of additional conventional clips for complete defect closure.

Complete closure of defects after colorectal ESD may effectively minimize adverse events 1 . Furthermore, Kubosawa et al. reported that suturing along the long axis of the defect may prevent strictures after duodenal ESD 2 . However, closure of large ESD defects is technically difficult because of slippage of the clip over the mucosa when apposition across a wide distance is required. Various closing methods have been reported 3 4 5 , all of which require additional preparation. This case report highlights the use of a novel re-openable endoclip with anchor prongs located in its jaws, which enables the closure of large ESD defects and facilitates the use of conventional clips after initial clip placement.

Endoscopy_UCTN_Code_TTT_1AQ_2AD

## Notes

Endoscopy E-Videos

https://eref.thieme.de/e-videos
*E-Videos* is an open access online section of the journal *Endoscopy* , reporting on interesting cases and new techniques in gastroenterological endoscopy. All papers include a high-quality video and are published with a Creative Commons CC-BY license. Endoscopy E-Videos qualify for HINARI discounts and waivers and eligibility is automatically checked during the submission process. We grant 100% waivers to articles whose corresponding authors are based in Group A countries and 50% waivers to those who are based in Group B countries as classified by Research4Life (see: https://www.research4life.org/access/eligibility/ ). This section has its own submission website at https://mc.manuscriptcentral.com/e-videos
